# Neutralizing antibodies against Simbu serogroup viruses in cattle and sheep, Nigeria, 2012–2014

**DOI:** 10.1186/s12917-018-1605-y

**Published:** 2018-09-10

**Authors:** Daniel Oluwayelu, Kerstin Wernike, Adebowale Adebiyi, Simeon Cadmus, Martin Beer

**Affiliations:** 1 Department of Veterinary Microbiology, University of Ibadan, Ibadan, Oyo State Nigeria; 2 Institute of Diagnostic Virology, Friedrich-Loeffler-Institut, Südufer 10, 17493 Greifswald - Insel Riems, Germany; 3 Department of Veterinary Public Health and Preventive Medicine, University of Ibadan, Ibadan, Oyo State Nigeria

**Keywords:** Schmallenberg virus, Simbu virus, Shamonda virus, Cattle, Sheep, ELISA, Neutralizing antibodies

## Abstract

**Background:**

Simbu serogroup viruses of the *Orthobunyavirus* genus (Family *Peribunyaviridae*) include teratogenic pathogens that cause severe economic losses, abortions, stillbirths and congenital abnormalities in ruminants worldwide. Although they were initially isolated from ruminants and *Culicoides* biting midges about five decades ago in Nigeria, there is no current information on their prevalence and geographical distribution despite reports of abortions and congenital malformations in the country’s ruminant population. Here, apparently healthy cattle and sheep obtained from eight states in the three major vegetation zones of Nigeria were screened for the presence of specific neutralizing antibodies against Schmallenberg virus (SBV), Simbu virus (SIMV) and Shamonda virus (SHAV).

**Results:**

Using a cross-sectional design, 490 cattle and 165 sheep sera were collected between 2012 and 2014 and tested by a commercial SBV ELISA kit which enables the detection of antibodies against various Simbu serogroup viruses. The seropositivity rates for cattle and sheep were 91.2% and 65.4%, respectively. In cattle, there was no association between ELISA seropositivity and vegetation zone. However, the prevalence of anti-Simbu serogroup antibodies was significantly higher in Ebonyi State compared to other states in the rainforest vegetation zone. The seroprevalence was significantly higher in sheep obtained from live animal markets compared to farms (OR = 5.8). Testing of 20 selected ELISA-positive sera by serum neutralisation test showed that all were positive for one or more of SBV, SIMV and SHAV with the highest titres obtained for SHAV. Antibodies to SBV or a closely related virus were detected in the Sudan savannah and rainforest zones, anti-SIMV antibodies were detected only in the rainforest zone, while anti-SHAV antibodies were found in the three vegetation zones.

**Conclusion:**

The findings of this study reveal that following the early isolation of Simbu serogroup viruses in Nigeria in the 1960s, members of this virus group are still circulating in the country. Specifically, SBV, SIMV and SHAV or closely related viruses infect cattle and sheep across the three vegetation zones of Nigeria suggesting that insect vector activity is extensive in the country. The exact vegetation zone where the animals became exposed to the viruses could, however, not be determined in this study.

## Background

The order *Bunyavirales* currently consists of more than 350 viruses that are distributed among 13 genera in nine families, thus making it one of the largest orders of RNA viruses. Of these genera, the *Orthobunyavirus* genus (family *Peribunyaviridae*) is the largest and most diverse with more than 170 viruses. The majority of these viruses are assigned to one of 18 serogroups, including the Simbu serogroup, based on serologic relatedness of complement fixing, hemagglutination inhibiting and neutralizing antibodies [1–3]. Members of this genus are arthropod-borne viruses (arboviruses) that are mostly transmitted by mosquitoes, sandflies or *Culicoides* biting midges, possess a tripartite RNA genome and share common genetic features but are serologically unrelated to viruses in other genera of the *Peribunyaviridae,* and many are pathogenic to humans and animals [4, 5].

In particular, the Simbu serogroup comprises at least 25 viruses that are currently divided into seven species, namely: Akabane virus (AKAV), Manzanilla virus, Oropouche virus, Sathuperi virus (SATV), Shamonda virus (SHAV), Shuni virus (SHUV) and Simbu virus (SIMV) [1, 2]. Several of these Simbu serogroup viruses are known to be teratogenic in ruminants [6] causing abortions, stillbirths and congenital abnormalities. While some members such as SHAV, SHUV, Sabo, and Sango viruses are less frequently examined, AKAV, Aino virus (AINV) and Schmallenberg virus (SBV) are the most studied in this serogroup [7–10].

Virus isolation or serological methods have been used to detect Simbu serogroup viruses in domestic animals, wildlife, mosquitoes and *Culicoides* from Africa, Asia, Australia, and the Middle East [11–19]. Although different assays including serum neutralization test (SNT), immunofluorescence (IF) assay and enzyme-linked immunosorbent assay (ELISA) have been used for the serologic detection of previous infections with these viruses [16, 20–22], specific detection of antibodies against them can be achieved by SNT [20].

Simbu serogroup viruses have been reported to cause severe economic losses to the livestock industry worldwide [23, 24]. However, information on their presence in Africa is still relatively scarce. In Nigeria, where the climate favours vector activity, early arboviral studies [7, 25] led to the isolation of Simbu serogroup viruses including SHAV, Sabo, Sango, SHUV and SATV viruses from cattle, goats and *Culicoides* biting midges. However, for about five decades there has been no information on the prevalence, geographical distribution and reproductive impact of these viruses despite reports of abortions, stillbirths and congenital malformations in the country’s ruminant population [26–28]. Recent studies based on commercial ELISAs to elucidate the role of Simbu serogroup viruses in the occurrence of reproductive disorders and congenital malformations among ruminants in Nigeria provided serologic evidence of AKAV, SBV or closely related viruses [19, 29]. However, because of the antigenic cross-reactivity that exists among Simbu serogroup viruses, the current study was conducted to investigate the presence of specific neutralizing antibodies against Schmallenberg, Simbu and Shamonda viruses in apparently healthy cattle and sheep obtained from eight states spread across the three major vegetation zones of Nigeria.

## Methods

### Study area

**Fig. 1:**
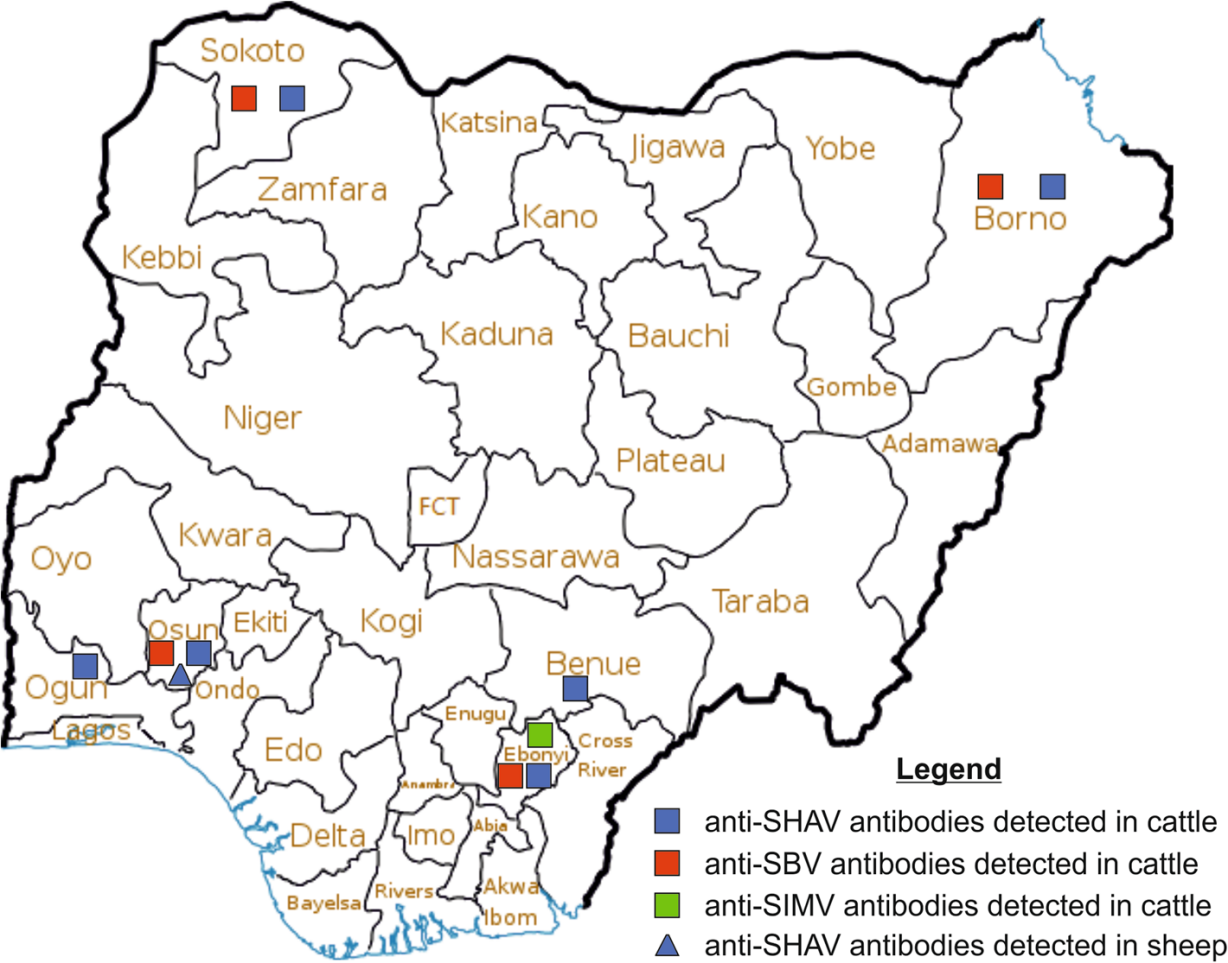
Map of Nigeria showing sample collection sites and distribution of samples that tested positive for antibodies against Schmallenberg virus (SBV), Simbu virus (SIMV) or Shamonda virus (SHAV). Source of the map: User:Gar3th [CC BY 3.0 (https://creativecommons.org/licenses/by/3.0)], from Wikimedia Commons (https://commons.wikimedia.org/wiki/Atlas_of_Nigeria#/media/File:Nigeria_states.png)

This study was carried out as part of recent investigations to determine the contribution of Simbu serogroup viruses to cases of reproductive disorders and congenital malformations in the Nigerian ruminant population. Cattle sera were collected from abattoirs, live animal markets, private/backyard farms or Fulani pastoralist herds located in eight states of Nigeria. These states include Borno (Northeast) and Sokoto (Northwest) located in the Sudan savannah vegetation zone and sharing international borders respectively with Chad and Niger Republic, two countries that are major suppliers of cattle to Nigeria. The other states, which serve as transit or sales points for cattle and sheep in their respective regions, are Benue (North-central) in the Guinea savannah zone, and Ebonyi (Southeast), Ogun, Osun, Oyo and Lagos (Southwest) in the rainforest zone. Sera from sheep were collected from live animal markets, private/backyard farms or Fulani herds located in Ogun, Osun, Oyo and Lagos States (Fig. 1). The exact origin of animals sampled at abattoirs and live animal markets in the North-central, Southeast and Southwest states could not be determined, but most of them were transported in trucks from the North-eastern and North-western regions of the country. Animals in the private/backyard farms were raised under the commonly practised semi-intensive system of management where they were fed on pastures or cut grasses during the day and kept in non-insect-proof sheds at night, while the Fulani herds grazed extensively from one location to another in their traditional manner. Thus, all the animals were exposed to insect vectors.

### Study design and sample collection

A cross-sectional design was used for this study. Serum samples were collected between May 2012 and April 2014 from 490 adult cattle in the eight states as follows: Borno (*n* = 84), Sokoto (*n* = 89), Benue (*n* = 83), Ebonyi (*n* = 67), Ogun (*n* = 30), Osun (*n* = 34), Oyo (n = 30) and Lagos (*n* = 73), while 165 sera from adult sheep were collected from the southwestern states of Ogun (*n* = 57), Osun (*n* = 70), Oyo (*n* = 25) and Lagos (*n* = 13). The animals were randomly selected at the individual study sites. The separated sera were stored at -20 °C until analysed.

### Serologic testing

#### Simbu serogroup enzyme-linked immunosorbent assay (ELISA)

All the 655 sera were initially screened for the presence of anti-Simbu serogroup antibodies using the ID Screen® Schmallenberg virus competition multi-species ELISA kit (IDvet, France), which detects antibodies against various Simbu serogroup viruses [30], according to the manufacturer’s instructions. For each serum sample, the competition percentage (S/N%) was calculated. Samples presenting S/N% ≤ 40%, 40% < S/N% ≤ 50%, and > 50% were considered positive, doubtful and negative, respectively.

#### Serum neutralization test (SNT)

A subset of 20 serum samples selected based on their very low S/N% values (i.e. high seropositivity) in the ELISA was analysed in microneutralization tests performed as previously described [31] against SBV, SIMV and SHAV. Briefly, two-fold dilutions of sera were prepared in Minimum Essential Medium (MEM), starting with 1:20. Fifty μl of MEM containing 100 TCID_50_ of SBV, SIMV or SHAV and 50 μl of the diluted sera were incubated in microtitre plates for 2 h. Thereafter, a BHK cell suspension (in 100 μl of MEM containing 10% foetal calf serum) was added and the microtitre plates were incubated for 3 days at 37 °C. Evaluation was done by assessment of the cytopathic effect. All sera were tested in three replicates and the titres were determined using the method described by Reed and Muench [32] and expressed as the 50% neutralizing dose per ml (ND_50_/ml). The samples included 15 cattle sera (three each from the Northeast, Northwest, North-central, Southeast and Southwest regions) and five sheep sera from the Southwest.

### Statistical analysis

Data were analyzed using GraphPad Prism version 5.01 (San Diego, USA). Differences in Simbu serogroup antibody seroprevalence between cattle and sheep, and female and male animals were evaluated using Chi-square (*χ*2) test. Seroprevalence data based on vegetation zones, source of animals and location/state were subjected to one-way ANOVA and subsequently to Tukey’s post-test for performing multiple comparisons. Statistical differences between all possible pairs of groups were assessed at *P* < 0.05.

## Results

### Simbu serogroup ELISA

Out of 490 cattle sera tested for anti-Simbu serogroup antibodies with the ELISA, 447 (91.2%), 20 (4.1%) and 23 (4.7%) were positive, doubtful and negative, respectively, while of the 165 sheep sera tested, 108 (65.4%), 11 (6.7%) and 46 (27.9%) were positive, doubtful and negative, respectively. In cattle, the prevalence of positive ELISA results was significantly higher in Ebonyi State compared to other states in the rainforest vegetation zone. Also, there were significant differences based on the origin of cattle tested (Table 1). Prevalence of anti-Simbu serogroup antibodies was significantly higher in sheep from live animal markets compared to farms (OR 5.8, 95% CI: 2.4–14.1), and in Ogun and Osun States relative to the other two states (Table 2).

**Table 1:** Results of the Simbu serogroup ELISA obtained from cattle sera

	Vegetation zone								Source			Sex		Total
	*Sudan savannah*		*Guinea savannah*	*Rainforest*										
	Sokoto	Borno	Benue	Ebonyi	Ogun	Osun	Oyo	Lagos	Live animal markets	Farms	Abattoirs	Female	Male	
No. sampled	89	84	83	67	30	34	30	73	326	47	117	294	196	490
Positive (%)	79 (88.8)	82 (97.6)	80 (96.4)	42 (62.7)	30 (100.0)	34 (100.0)	30 (100.0)	70 (95.9)	286 (87.7)	47 (100.0)	114 (97.4)	264 (89.8)	183 (93.4)	447 (91.2)
Doubtful (%)	–	2 (2.4)	2 (2.4)	13 (19.4)	–	–	–	3 (4.1)	17 (5.2)	–	3 (2.6)	14 (4.8)	6 (3.1)	20 (4.1)
Negative (%)	10 (11.2)	–	1 (1.2)	12 (17.9)	–	–	–	–	23 (7.1)	–	–	16 (5.4)	7 (3.6)	23 (4.7)

**Table 2:** Results of the Simbu serogroup ELISA obtained from sheep sera

	No. sampled	Positive (%)	Doubtful (%)	Negative (%)
State				
Ogun	57	50 (87.7)	2 (3.5)	5 (8.8)
Osun	70	51 (72.9)	6 (8.6)	13 (18.6)
Oyo	25	4 (16.0)	1 (4.0)	20 (80.0)
Lagos	13	3 (23.1)	2 (15.4)	8 (61.5)
Sex				
Female	91	56 (61.5)	7 (7.7)	28 (30.8)
Male	74	52 (70.3)	4 (5.4)	18 (24.3)
Source				
Live animal markets	64	55 (85.9)	2 (3.1)	7 (10.9)
Farms	101	53 (52.5)	9 (8.9)	39 (38.6)
Total	165	108 (65.4)	11 (6.7)	46 (27.9)

### Serum neutralization tests

Antibodies against SBV, SIMV and SHAV or closely related viruses were detected in six of the eight states (Fig. 1), with variable neutralizing antibody titres in cattle and sheep (Table 3). All the 20 sera tested were positive for antibodies against at least one of the three Simbu serogroup viruses SBV, SIMV and SHAV which were included in the present study. Of these 20 sera, 7 (35.0%), 2 (10.0%) and 20 (100.0%) were positive for SBV, SIMV and SHAV antibodies, respectively, while 6 (30.0%), 1 (5.0%) and 1 (5.0%) were positive for a combination of SBV and SHAV, SIMV and SHAV, and SBV, SIMV and SHAV antibodies, respectively (Table 3).

**Table 3:** Neutralizing antibody titres against the Simbu serogroup viruses Schmallenberg virus (SBV), Simbu virus (SIMV) and Shamonda virus (SHAV) in the tested Nigerian cattle and sheep sera

No.	Species	Breed	Sex	Source	State	Neutralising titre (ND_50_/ml)		
						SBV	SIMV	SHAV
1	Cattle	Red Bororo	Female	Abattoir	Borno	1/57	neg.	1/180
2	Cattle	Red Bororo	Female	Abattoir	Borno	1/36	neg.	1/180
3	Cattle	Red Bororo	Female	Abattoir	Borno	1/57	neg.	1/180
4	Cattle	Sokoto Gudali	Female	Abattoir	Sokoto	1/71	neg.	1/143
5	Cattle	White Fulani	Female	Abattoir	Sokoto	neg.	neg.	1/180
6	Cattle	White Fulani	Female	Abattoir	Sokoto	1/113	neg.	1/90
7	Cattle	White Fulani	Male	Abattoir	Benue	neg.	neg.	1/904
8	Cattle	Red Bororo	Female	Abattoir	Benue	neg.	neg.	1/226
9	Cattle	Red Bororo	Female	Abattoir	Benue	neg.	neg.	1/36
10	Cattle	White Fulani	Male	Abattoir	Ebonyi	neg.	neg.	1/90
11	Cattle	Sokoto Gudali	Female	Abattoir	Ebonyi	1/57	1/113	1/71
12	Cattle	Sokoto Gudali	Male	Abattoir	Ebonyi	neg.	1/143	1/71
13	Cattle	Sokoto Gudali	Male	Farm	Ogun	neg.	neg.	1/57
14	Cattle	White Fulani	Male	Farm	Osun	neg.	neg.	1/113
15	Cattle	White Fulani	Female	Farm	Osun	1/36	neg.	1/36
16	Sheep	Ouda	Male	Live animal market	Osun	neg.	neg.	1/226
17	Sheep	Yankassa	Male	Live animal market	Osun	neg.	neg.	1/45
18	Sheep	Yankassa	Female	Live animal market	Osun	neg.	neg.	1/143
19	Sheep	Balami	Female	Live animal market	Osun	neg.	neg.	1/71
20	Sheep	Balami	Male	Live animal market	Osun	neg.	neg.	1/71

neg. = < 1/20 ND_50_/ml

The geographic distribution of the 20 SNT-positive sera showed that antibodies reacting with SBV were detected in Borno and Sokoto States (Sudan savannah zone) as well as in Ebonyi and Osun States (rainforest zone), antibodies to SIMV were found only in Ebonyi State (rainforest zone), while antibodies to SHAV were found in Borno, Sokoto, Benue, Ebonyi, Osun and Ogun States, representing the three vegetation zones (Fig. 1). Information on the breed, sex and source of the SNT-positive sera are shown in Table 3.

## Discussion

Simbu serogroup viruses are generally believed to be endemic in Africa. Apart from their initial isolation in Nigeria several decades ago, antibodies to several viruses in this serogroup were also detected in the 1970s in Kenya, South Africa and Nigeria [7, 12, 13, 25]. Although the ELISA-based detection of antibodies against SBV, a newly emerged Simbu serogroup virus, was recently reported in domestic ruminants in Mozambique and Tanzania [17, 18], current knowledge about the occurrence, distribution and spread of these viruses in Nigeria is scarce. Only recently, ruminant sera were screened by commercially available SBV and AKAV antibody ELISAs in small-scale pilot studies [19, 29]. In the present follow-up study a more comprehensive sample set including bovine and ovine sera was collected. In addition, all three major vegetation zones of Nigeria were integrated in the study. Since cross-reactivity with various closely related viruses might occur when using S-segment based commercial SBV ELISAs [22], the more specific SNT was performed for further characterisation.

The results of this study show that 84.7% (555/655) of the animals tested had antibodies against Simbu serogroup viruses based on the commercial ELISA test. This high seroprevalence rate suggests that a large proportion of Nigeria’s cattle and sheep population had been exposed to infection with SBV-related or other Simbu serogroup viruses. However, since it had been reported that antibodies against viruses in this serogroup frequently cross-react with more than one other member of the group [2, 18, 33], we performed SNT on the ELISA-positive serum samples for serologic confirmation. Our findings revealed that Simbu serogroup viruses continue to infect livestock in Nigeria and that at least three of them, namely SBV, SIMV and SHAV, were circulating in the country during the 2012–2014 or previous seasons. The detection of neutralizing antibodies to SBV and SHAV in cattle from Sokoto and Borno States (Sudan savannah zone) and the presence of neutralizing antibodies to SBV, SIMV and SHAV in one serum sample from Ebonyi State (rainforest zone) highlight the existence of either mixed infections with these viruses or antigenic cross-reactivity among them. Another possible explanation for this finding could be that the animals were infected with other Simbu serogroup viruses (not SBV, SIMV or SHAV) that cross-react to a certain extent with these three viruses. Such viruses, including AKAV and AINOV, are associated with epizootics of congenital malformations in ruminants and have been isolated from *Culicoides* biting midges in Africa, Australia, the Middle East and Asia [12, 34–36].

Additionally, it is noteworthy that despite being positive in the commercial ELISA test sold for anti-SBV antibody detection, only anti-SHAV antibodies were detected in cattle sera from Benue State (Guinea savannah zone) and sheep sera from Osun State (rainforest zone). This finding further corroborates reports of the existence of cross-reactivity among the Simbu serogroup viruses. Moreover, the fact that only Ebonyi State had cattle that were seropositive for SIMV suggests limited circulation of the virus although a larger sample size covering almost all the states of the country might be needed to verify this. Overall, while the seroprevalence for SIMV and SBV were low (2/20, 10.0%) to moderate (7/20, 35.0%) respectively, that of SHAV was exceptionally high (20/20, 100.0%).

The detection of neutralizing antibodies against SBV, SIMV and SHAV as well as the distribution of seropositive animals (Fig. 1) indicates that these viruses or related Simbu serogroup viruses infect cattle and sheep across the different vegetation zones of Nigeria and that the activity of insect vectors, most likely *Culicoides* biting midges, is widespread in the country. The exact vegetation zone where the animals became exposed to the viruses could, however, not be determined in this study. Further studies will be necessary to isolate and characterize Simbu serogroup viruses circulating in the Nigerian ruminant population and in the insect vectors responsible for their transmission. In addition, the possible negative impact of these viruses on reproductive performance of cattle and sheep in Nigeria needs to be investigated.

## Conclusions

The findings of the present study reveal that following the early isolation of Simbu serogroup viruses in Nigeria in the 1960s, members of this virus group are still circulating in the country. Specifically, SBV, SIMV and SHAV or closely related viruses infect cattle and sheep across the three vegetation zones of Nigeria suggesting that activity of competent insect vectors is extensive in the country.

## Abbreviations

AINV: Aino virus
AKAV: Akabane virus
ELISA: enzyme-linked immunosorbent assay
IF: immunofluorescence
SATV: Sathuperi virus
SBV: Schmallenberg virus
SHAV: Shamonda virus
SHUV: Shuni virus
SIMV: Simbu virus
SNT: serum neutralisation test
